# Hereditary Hypophosphatemic Rickets with Hypercalciuria (HHRH) Presenting with Genu Valgum Deformity: Treatment with Phosphate Supplementation and Surgical Correction

**DOI:** 10.1155/2020/1047327

**Published:** 2020-07-09

**Authors:** Juan M. Colazo, Seth A. Reasoner, Ginger Holt, Marie C. M. Faugere, Kathryn M. Dahir

**Affiliations:** ^1^Department of Biomedical Engineering, Vanderbilt University, Nashville, TN, USA; ^2^Medical Scientist Training Program, Vanderbilt University School of Medicine, Nashville, TN, USA; ^3^Department of Pathology, Microbiology and Immunology, Vanderbilt University Medical Center, Nashville, TN, USA; ^4^Department of Orthopedics, Vanderbilt University Medical Center, Nashville, TN, USA; ^5^Division of Nephrology, Bone and Mineral Metabolism, University of Kentucky, Albert B. Chandler Medical Center, Lexington, KY, USA; ^6^Division of Diabetes, Endocrinology and Metabolism, Vanderbilt University Medical Center, Nashville, TN, USA

## Abstract

We describe a case of hereditary hypophosphatemic rickets with hypercalciuria (HHRH) in a 32-year-old female with short stature, chronic pathologic genu valgum deformity, and knee pain who was referred to endocrinology clinic after previous inconclusive workups. We present imaging spanning 10 years of untreated disease. Biochemical studies showed hypophosphatemia with undetectable fibroblast growth factor 23 (FGF23.) Renal ultrasound revealed bilateral medullary nephrocalcinosis despite no apparent hypercalciuria. Due to concern for HHRH, genetic testing was performed that determined this patient to be homozygous in the *SLC34A3* gene for a previously described missense variant (c.1402C > T, p.Arg468Trp). There was no known family history of rickets. A bone biopsy with metabolic studies was performed for diagnostic and prognostic reasons. The histopathological findings along with tetracycline uptake studies were consistent with a diagnosis of HHRH. Treatment with phosphorous supplementation and surgical correction of her valgum deformity resulted in resolution of pain, but no change in bone histomorphometry.

## 1. Introduction

Hereditary hypophosphatemia rickets with hypercalciuria (HHRH) is an extraordinarily rare autosomal recessive disorder with an estimated prevalence of 1/250,000 [1]. Variants in the SLC34A3 gene encoding a sodium-dependent phosphate transporter (NaPi-IIc) have been implicated as responsible for this disease. The NaPi-IIc transporter is highly expressed in the nephron's proximal tubules, and its dysfunction results in phosphate wasting disorder.

Here, we report a case of HHRH presenting as a genu valgum deformity with pain in a 32-year-old female with no fracture history and unremarkable family history for metabolic bone disease. We review the pathophysiology, diagnosis, and treatment of HHRH and contrast HHRH and other phosphate wasting disorders.

## 2. Case Presentation

**Figure 1:**
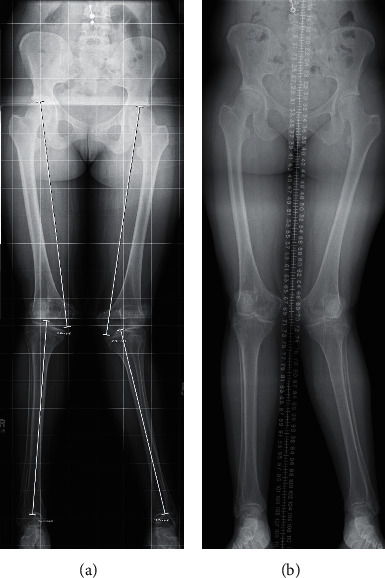
Radiographs of lower extremity genu valgus. (a) At age 22, ten years prior to the current presentation, radiography displays genu valgum of the left knee measuring 17° with a hypoplastic lateral femoral condyle. (b) At current presentation, at age 32, redemonstration of genu valgum of the left knee measuring 23° with narrowing of the joint compartments at the knees and osteochondral irregularity of the talar dome on the right.

A 32-year-old Caucasian female presented to endocrinology clinic with severe left knee pain and a significant genu valgum deformity bilaterally. The patient reported being told that “she may have had rickets” at the age of 9 but was lost to follow-up. Ten years prior to this current presentation, she presented at the age of 22 with painful trochanteric bursitis. During this clinic appointment, X-ray images showed a significant genu valgum deformity at a Q- angle of 17° (Figure 1). Deformity correction was suggested at this time but was not performed. At that time, no endocrinologic workup was performed.

Now, at the age of 32, a complete medical history was obtained. She is currently taking over-the-counter vitamin D3, but no calcium supplementation. She is 155 cm (58.5 kg and BMI 23.79 kg/m^2^), her mother is 173 cm, and her father is 188 cm. She has no siblings or children. She has no fracture or abnormal dentition history. There is no family history of rickets or other metabolic bone disease.

**Figure 2:**
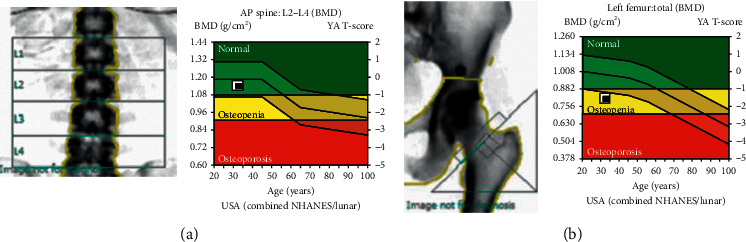
Bone mineral density (BMD) scans (current presentation). (a) The BMD within the lumbar spine is 1.140 g/cm^2^ which corresponds to a *Z*-score of −0.4. (b) The BMD within the left total hip is 0.807 g/cm^2^ which corresponds a *Z*-score of −1.4. The BMD within the left femoral neck is 0.819 g/cm^2^ which corresponds to a *Z*-score of −1.3.

**Figure 3:**
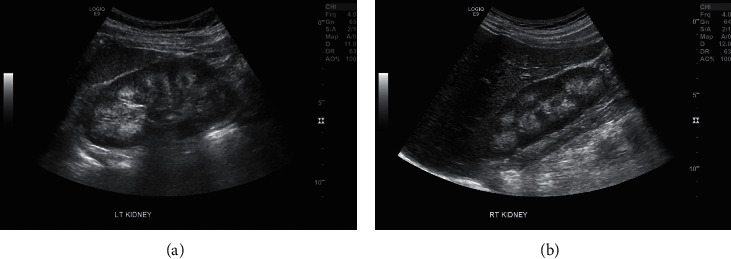
Renal ultrasound (current presentation). (a) Left kidney measuring 11.7 cm in length. (b) Right kidney measuring 11.5 cm in length. Both kidneys have homogenous cortical echoes. The renal pyramids are hyperechoic consistent with bilateral medullar nephrocalcinosis.

On physical examination, there is marked valgus alignment bilaterally, with left being significantly more prominent. Gait examination showed her left knee knocking into her right leg. X-ray scans were performed that showed a Q-angle of 23°, worsened from her presentation 10 years prior (Figure 1). Bone mineral density scans showed osteopenia of the left femoral head with normal lumbar spine density (Figure 2). Pertinent laboratory values displayed hypophosphatemia, elevated total vitamin D and 1,25-dihydroxyvitamin D, undetectable fibroblast growth factor 23 (FGF23), and low parathyroid hormone (Table 1). Twenty-four-hour urine showed inappropriately normal phosphate excretion and normal urine calcium. A renal ultrasound showed bilateral medullary nephrocalcinosis, without calculi (Figure 3). HHRH was suspected due to her described biochemistry and the presence of bilateral renal calcinosis. Thus, genetic testing was pursued, and this patient was determined to be homozygous in the *SLC34A3* gene for a previously described missense variant (c.1402C > T, p.Arg468Trp) (NM_080877.2) [2, 3]. Due to these findings, she was started on phosphorous supplementation (K-Phos Neutral 250 mg TID AC).

**Figure 4:**
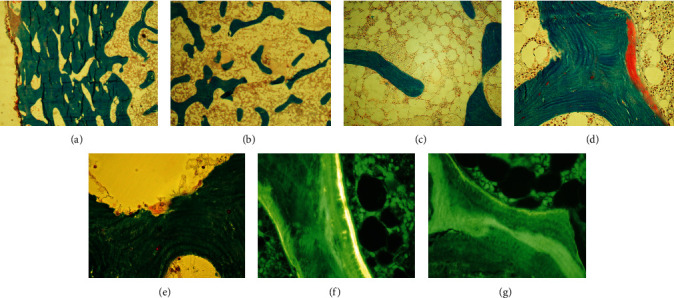
Iliac crest bone biopsy prior to phosphorus supplementation. Modified Masson–Goldner trichrome (a)–(e). High porosity of the cortical bone (a) and decreased cancellous bone volume (b). Low bone turnover and mild hypocellularity of bone marrow (c). Rare osteoid seam of normal thickness (d) and few osteoclasts (e). Fluorescent light microscopy: few tetracycline label (f) and evidence of a previous oxytetracycline label (g).

A right iliac crest bone biopsy was performed and sent for metabolic bone studies prior to the initiation of phosphorus. Histopathological findings included high porosity of the cortical bone, decreased cancellous bone, and decreased osteoid surface (Figure 4). Fluorescent light microscopy with tetracycline labelling showed a decrease in the mineralizing surface. Some double labels were seen in the subcortical bone. The distance between double labels was in the low-normal range. These results show previous weakly diffuse tetracycline deposition (Figures 4(e) and 4(f)), determined to be most likely due to her recent prescription of doxycycline for facial acne therapy which was stopped in advance of the biopsy. In summary, the metabolic bone biopsy showed low bone volume with reduced turnover of cancellous bone without evidence of osteomalacia.

**Figure 5:**
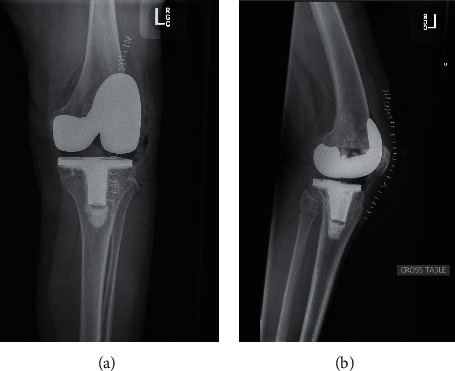
Postoperative radiographs. Postoperative imaging following corrective left total knee arthroplasty: anterior-posterior (a) and lateral views (b).

**Figure 6:**
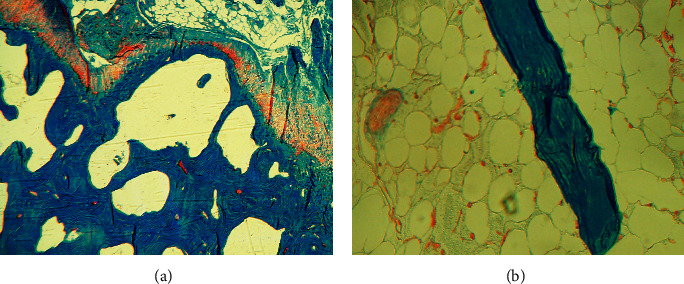
Intraoperative surgical biopsy after phosphorus supplementation. Modified Masson–Goldner trichrome of a femur specimen obtained at the time of knee arthroplasty displayed thin cortical bone and high porosity (a). Thin trabecula and adipose bone marrow (b). No significant change in comparison with her prior biopsy.

Since the diagnosis of HHRH, she continued to receive phosphorous supplementation; meanwhile, vitamin D was stopped. Her abnormal laboratory values have resolved (Table 1). Her left knee pain has improved significantly. Six months after starting phosphorus supplementation, she underwent an uncomplicated left total knee arthroplasty (TKA) leading to gait correction (Figure 5). Biopsy at the time of surgery showed thin cortical bone and high porosity without evidence of osteomalacia, not significantly changed in comparison with her previous biopsy (Figure 6).

## 3. Discussion

The patient in this case initially presented at the age of 9 and received an ambiguous diagnosis of “rickets.” She did not receive a definitive diagnosis and begin correct treatment until 20 years later. Like other cases in the literature, our case highlights the protracted diagnostic journey for many of these patients with incorrect or uncertain diagnoses along the way [4]. This patient had presented in childhood with leg pain and genu valgum deformity. To understand the differential diagnosis for deformities such as genu valgum or “knock knee,” one must first understand the natural history of the alignment of the lower limbs [5]. In newborns and infants, “bowlegs” (bilateral genu varus) are physiologic. These become straight by around 18–24 months. By 2 or 3 years, “knock knee” (physiologic genu valgus) develops with an average Q-angle of about 12°. By 7 years, spontaneous correction usually occurs to the physiologic alignment of adult valgus (mean of about 7° in females and 5° in males) and anything >12° is considered pathological. If valgus is sustained past this point, it is considered pathological and can cause significant distress, referred pain, and pressure, causing trochanteric bursitis as seen in this case.

A correct workup for these deformities is essential. If a patient is misdiagnosed, surgical correction may fail and cause healing difficulties. The differentialdiagnosis for bilateral genu valgum includes physiologic, renal osteodystrophy (renal rickets), skeletal dysplasia, Morquio syndrome, spondyloepiphyseal dysplasia, chondroextodermal dysplasia, and metabolic disorders including HHRH. The differential diagnosis for unilateral genu valgum includes physeal injury from trauma, infection, or vascular insult, proximal metaphyseal tibia fracture, benign tumors (fibrous dysplasia, osteochondromas, and Ollier's disease), among others [6]. Our patient had bilateral valgus, with unilateral predominance. Thus, a metabolic bone disorder was most likely.

Tieder et al. were the first to describe the clinical phenotype of HHRH in a consanguineous Bedouin family [7]. Since then, case reports have been published from individuals from a variety of ethnicities, leading to the realization that HHRH is likely an underrecognized cause of rickets and osteomalacia [8]. In fact, patients misdiagnosed with osteoporosis have been later found to have HHRH [4]. Patients usually present in the first years of life with rickets, bone pain, short stature, and lower extremity deformities. Patients with this condition can also develop recurrent nephrolithiasis and/or medullary nephrocalcinosis. The classically published biochemical phenotype includes hypophosphatemia, phosphaturia, hypercalciuria, elevated 1,25-dihydroxyvitamin D levels, and suppressed PTH levels. We will discuss each of these electrolyte and endocrinologic abnormalities in more detail below.

HHRH is caused by variants in the *SLC34A3* gene which codes for the proximal tubular transporter NaPi-IIc. NaPi-IIc is one of three known phosphate transporters in the proximal tubule: NaPi-IIa, NaPi-IIc, and PiT_2_ [9]. NaPi-IIc reabsorbs divalent phosphate (HPO_4_^2−^) from the filtrate in exchange of sodium ions. Variants in *SLC34A3*/NaPi-IIc lead to impaired phosphate reabsorption and thus phosphate wasting in the urine. At least 40 separate pathogenic variants in *SLC34A3* have been identified [1]. The inheritance pattern is autosomal recessive and often caused by compound heterozygotic variants. Interestingly, carriers of a single *SLC34A3* variant display an intermediate phenotype with increased frequency of renal calculi compared to the general population [10]. This patient's parents, presumed to be carriers, had no history of nephrolithiasis and were of normal stature.

Phosphate wasting in HHRH leads to elevation of 1,25-dihydroxyvitamin D levels, a key clue differentiating HHRH from other phosphate wasting disorders. Elevated 1,25-dihydroxyvitamin D levels in these patients cause increased calcium absorption from the gastrointestinal tract which results in transient hypercalcemia, suppressed PTH levels, and a compensatory hypercalciuria [11]. Occasionally, an incidentally discovered hypercalcemia can trigger a diagnostic workup leading to the diagnosis of HHRH. The reduction in PTH-dependent calcium-reabsorption in the distal renal tubules ultimately leads to nephrolithiasis and/or nephrocalcinosis. Another differentiating feature between HHRH and other phosphate wasting disorders is the low levels of fibroblast growth factor 23 (FGF23) in HHRH. The normal physiologic function of FGF23 is the regulation of phosphate concentration in the blood. FGF23 is secreted by osteocytes when 1,25-dihydroxyvitamin D levels are elevated. FGF23 then acts on the kidneys by decreasing the expression of NaPi-IIc [12]. In turn, FGF23 decreases the reabsorption of phosphate, causing phosphate wasting. In addition, recent studies have shown that FGF23 may also suppress 1-alpha-hydroxylase, reducing its ability to activate vitamin D and therefore impairing calcium absorption and homeostasis [13].

In evaluating hypophosphatemia, the patient's levels of vitamin D, FGF23, and PTH are particularly useful. Vitamin D levels are elevated in HHRH, whereas they are low or inappropriately normal in conditions of FGF23 excess. Vitamin D deficiency, however, can mask laboratory features of HHRH and therefore must be assessed independently [8, 14, 15]. Hypophosphatemic disorders with FGF23 excess are more common than HHRH and are caused by variants directly affecting FGF23 and its metabolism. For example, X-linked hypophosphatemia is caused by variants in *PHEX* which is an inactivator of FGF23 and is approximately ten times more common than HHRH [16, 17].

Treatment of FGF23-mediated phosphate wasting involves phosphate and vitamin D supplementation. In conditions of FGF23 excess, an emerging treatment option is Burosumab, an anti-FGF23 IgG1 monoclonal antibody, which has been recently approved for use in X-linked hypophosphatemia [18]. Since HHRH is an FGF23-independent disorder, Burosumab would be ineffective. Currently, the only treatment for HHRH is long-term phosphorous supplementation which can singularly cause remission and rapid correction of bone mineralization capacity. Meanwhile, the addition of active vitamin D metabolites can cause symptom deterioration and worsening of hypercalciuria and nephrolithiasis [14, 19]. Ultimately, HHRH should be treated with phosphorus supplementation for at least 6 months before corrective surgery (e.g., osteotomy and TKA) is pursued to ensure that bone mineralization is no longer compromised. If not, poor bone healing and mineralization may occur, causing further deficits that may be irreversible. Ideally, identification of HHRH in childhood might prevent the need for surgical correction later in life. The variable clinical presentations of HHRH, however, may not be recognized until after childhood.

The constellation of symptoms in HHRH can be variable in their onset and severity. Patients presenting in childhood generally have rickets and/or nephrolithiasis. In adulthood, nephrolithiasis and bone pain are the most common presenting symptoms. The presence of renal calcifications is more uncommon and is often attributed to inappropriate treatment of HHRH with vitamin D. The variability in the age of presentation is due to incomplete penetrance from various variants and environmental factors [15]. Serum phosphate is sensitive to dietary intake of phosphate. And, in turn, the compensatory endocrinologic mechanisms lead to fluctuating laboratory values [19, 20]. In this case, the renal ultrasound demonstrating nephrocalcinosis was particularly pivotal in the diagnosis due to the equivocal urine calcium measurement. Given the lengthy differential diagnosis for phosphate wasting, genetic analysis is generally considered necessary for a conclusive diagnosis of HHRH.

## 4. Conclusions

Herein, we presented the first reported case of surgical correction of a genu valgus deformity caused by hereditary hypophosphatemic rickets with hypercalciuria (HHRH). HHRH is an underidentified cause of rickets and osteomalacia and may be misdiagnosed as osteoporosis. We reviewed phosphate wasting disorders and their distinguishing features.

## Figures and Tables

**Table 1:** Laboratory values.

Lab test (reference value)	At presentation	After 6 months of phosphorus supplementation
Phosphorus (2.3–4.7 mg/dL)	1.7	2.5
Calcium (8.5–10.5 mg/dL)	9.6	8.9
Creatinine (0.57–1.11 mg/dL)	0.82	0.83
Total vitamin D (25–80 ng/mL)	85	56
1,25-Dihydroxy vitamin D (19.9–79.3 pg/mL)	154	126
FGF23 (≤180)	<50	—
Bone-specific alkaline phosphate (premenopausal female: 4.5–16.9 *μ*g/L)	12.1	12.7
Parathyroid hormone (16–77 pg/mL)	9	16
Urine phosphorus (400–1,300 mg/24 hr)	777^*∗*^	—
Urine calcium (100–300 mg/24 hr)	155	—

^*∗*^In patients with hypophosphatemia, urinary phosphate excretion above 100 mg/day or a fractional excretion, F_E_PO_4_, above 5 percent is indicative of renal phosphate wasting.

## Data Availability

Information and data can be accessed from the submitting author (juan.m.colazo@vanderbilt.edu) or the corresponding author (kathryn.dahir@vumc.org) via email.
